# Engaging Service Users in Co‐Designing a Nurse‐Led Weight Talk Tool for Low‐ and Medium‐Secure Mental Health Inpatient Settings: A Participatory Intervention Development Study

**DOI:** 10.1111/inm.70346

**Published:** 2026-09-17

**Authors:** Novia K. N. Mak, Bridget Dibb, Nicholas Stokes, Emerita Elizabeth Alexandra Barley, Magdalena Zasada

**Affiliations:** ^1^ School of Health Science, Faculty of Health and Medical Sciences University of Surrey Guildford UK; ^2^ School of Psychology Elizabeth Fry Building (AD) University of Surrey Surrey UK; ^3^ Research Department St. Bernard's Hospital Southall UK

**Keywords:** cooperative behaviour, health communication, mental health services, patient participation, psychiatric nursing

## Abstract

Approximately 80% of individuals in secure mental health units are overweight or obese, yet nurses often lack practical guidance on how to initiate and sustain weight‐related conversations in these complex settings. This study aimed to co‐design an evidence‐based, nurse‐led weight talk tool with service users and nursing staff, and to evaluate its acceptability and usability in low‐ and medium‐secure inpatient care. A multi‐stage, iterative design was undertaken. Initial evidence informed development, followed by a person‐centred co‐design process involving service users and staff through workshops, structured discussions and repeated feedback. The tool was subsequently evaluated using a think‐aloud approach with 19 nurse–service user pairs, enabling real‐time exploration of usability, understanding, and emotional responses. Co‐design produced a structured, visually engaging tool tailored to the sensitivities and constraints of secure mental health settings. Service users and staff shaped key refinements, including simplified language, removal of potentially stigmatising elements, enhanced visual design, and the inclusion of structured opening and closing components to support psychologically safe conversations. Evaluation findings demonstrated high acceptability and usability. Service users valued the respectful, collaborative approach, while nurses reported increased confidence in addressing weight‐related issues. These findings highlight the value of co‐design as a method for developing acceptable, contextually relevant interventions for sensitive clinical topics. Embedding collaborative approaches within mental health nursing practice may strengthen therapeutic engagement and support more effective, person‐centred care across diverse international settings.

## 1 Introduction

People residing in secure mental health inpatient settings experience disproportionately high rates of overweight and obesity. International evidence consistently reports elevated cardiometabolic risk and significantly reduced life expectancy among people living with severe mental illness, particularly those prescribed antipsychotic medication (Gronholm et al. 2021; Pedersen 2022). These disparities are observed across forensic and secure services in the United Kingdom, Australia, Europe and North America, highlighting obesity as a global physical health inequity within mental health care. For mental health nurses, addressing lifestyle‐related health concerns is therefore both a clinical responsibility and a matter of health equity (Attala et al. 2022).

In the UK, this issue is situated within broader preventative health policy. A prime driver of this is the National Health Service (NHS) ‘Making Every Contact Count’ (MECC) framework, which encourages healthcare professionals to use routine, opportunistic interactions to support patients in making positive lifestyle changes, particularly around physical activity, healthy diet and weight management (Public Health England 2016). For individuals with severe mental illness (SMI) residing in inpatient wards, who experience profound physical health inequalities and a reduced life expectancy, the mandate to deliver brief, preventative physical health interventions is vital. However, despite strong international policy emphasis on physical health monitoring and health promotion in mental health services (NHS England 2024), translating these national policy expectations into daily practice within forensic psychiatric wards remains a persistent challenge (Canning et al. 2026). Initiating and sustaining weight‐related conversations in secure settings remains challenging. Secure environments must balance therapeutic engagement with risk management and safety priorities. Findings from the qualitative phase of this research (manuscript under review) identified concerns about autonomy, consent, medication‐related weight gain, limited food choice and the potential impact of discussing weight within constrained environments. These findings align with international literature describing tensions between physical health promotion and custodial or security demands (Holley et al. 2020; Hurley et al. 2022; Rogers et al. 2021). However, there remains limited evidence describing how nurses can structure and deliver weight‐related conversations in ways that are both person‐centred and feasible within secure inpatient systems.

Interventions developed using top‐down approaches often fail to gain traction in restrictive clinical contexts because they insufficiently account for lived experience, ward culture and relational dynamics (Johnson et al. 2018). Co‐design offers a participatory alternative. By actively involving service users and clinicians in shaping intervention content, structure and delivery, co‐design seeks to produce tools that are contextually relevant, acceptable and sustainable (Silvola et al. 2023; Slattery et al. 2020). Within mental health nursing internationally, co‐design is increasingly recognised as a mechanism for embedding person‐centred care and recovery‐oriented values into intervention development (NHS England 2024).

This paper focuses specifically on the co‐design process used to develop a Nurse‐Led Weight Talk Tool—a structured yet flexible resource intended to guide respectful, collaborative weight‐related conversation in low‐ and medium‐secure inpatient units.

## 2 Background

Co‐design is increasingly recognised in mental health research as an approach that enhances the acceptability, relevance and sustainability of interventions by embedding lived experience within development processes (Richmond et al. 2023; Slattery et al. 2020). Beyond consultation, co‐design involves structured collaboration between researchers, clinicians, and people with lived experience, grounded in principles of inclusivity, shared decision‐making, and mutual respect (Pinfold et al. 2025). Within complex clinical systems, such approaches have been shown to strengthen engagement, contextual fit and implementation potential (Schouten et al. 2022).

Despite its growing prominence in mental health service innovation, detailed reporting of co‐design processes within research, particularly in secure inpatient settings, remains comparatively limited (McAllister et al. 2021). Existing studies across forensic and severe mental illness contexts demonstrate its potential to enhance intervention acceptability and sustainability (Anttila et al. 2025; Carswell et al. 2023). In particular, work evaluating the implementation and fidelity of MECC‐aligned weight management initiatives (such as the ‘A Weight Off Your Mind’ regional strategy) has demonstrated that while staff training can improve confidence in implementation, systemic and environmental barriers often hinder sustained practice (Kemp et al. 2024). Furthermore, qualitative evidence exploring service users' perspectives of weight management conversations in mental health wards highlights a critical tension: while service users highly value proactive physical health support, they frequently encounter a lack of clear information regarding medication‐related weight gain and stress that these sensitive conversations must be delivered within strong, empathetic therapeutic relationships (Kemp et al. 2025). This underscores the reality that fewer studies provide transparent accounts of how co‐design operates in ethically sensitive, restrictive environments where relational care, organisational constraint and power dynamics intersect. Addressing this gap is essential to advancing methodological rigour and transferability in secure mental health nursing research, ensuring that brief, opportunistic interventions like MECC are co‐designed to align with the unique relational needs of both service users and staff.

To support structured intervention development, this study was additionally informed by the Person‐Based Approach (PBA), which emphasises understanding how individuals engage with interventions in real‐world contexts to optimise usability and implementation (Yardley et al. 2015, 2021). The PBA complements co‐design by providing systematic mechanisms for translating experiential insights into clearly articulated intervention components.

In the UK, co‐design is closely aligned with the Patient and Public Involvement (PPI), which provides governance and ethical structures for meaningful collaboration in research (McCoy et al. 2018). Comparable participatory standards are increasingly embedded within research governance frameworks internationally. In this study, PPI principles were integrated across early intervention development phases to ensure transparent, inclusive and contextually grounded engagement.

Accordingly, this study addressed the following research question:

How can co‐design and PPI sessions inform the development of a nurse‐led weight management intervention for secure inpatient settings?

## 3 Methods

### 3.1 Study Design and Co‐Design Structure

This study employed a multi‐stage, mixed‐methods design underpinned by co‐design and Public and Patient Involvement (PPI). The study comprised four sequential components: (1) a systematic literature review; (2) qualitative interviews with service users and nursing staff to explore barriers and enablers to weight management discussions in secure inpatient care (reported elsewhere); (3) development of an initial Nurse‐Led Weight Talk Tool informed by evidence, theory and experiential insight; and (4) refinement and acceptability testing using think‐aloud methods.

PPI and co‐design, which are the focus of this paper, were embedded throughout all study phases to inform research questions, study design, interpretation and intervention development. Engagement activities were organised into six iterative rounds mapped onto the first six stages of the INVOLVE framework, progressing from project planning and prioritisation to tool testing and dissemination.

Each round used tailored engagement methods: structured group discussions facilitated with topic guides and visual prompts, Nominal Group Technique (NGT) for idea generation and prioritisation, and think‐aloud usability testing in paired formats. Sessions were facilitated with attention to psychological safety and adapted to accommodate operational constraints such as ward routines, staffing and participant availability. PPI feedback informed both the broader research design (e.g., refining questions, recruitment strategies, and ethical procedures) and the specific content, structure and design of the tool.

This structured co‐design approach ensured that both the research and the resulting intervention were grounded in lived experience and responsive to the practical, ethical and relational realities of secure mental health nursing practice. By embedding participatory methods throughout, the study strengthened the relevance, feasibility and acceptability of the intervention while promoting shared ownership among service users and staff.

### 3.2 Setting and PPI Contributors

The study took place within low‐ and medium‐secure inpatient mental health units in a large NHS Trust in England. Contributors included the following.

#### 3.2.1 Professionals

A total of 46 professional contributors participated in PPI activities across early study rounds. This comprised a core multi‐disciplinary group from the Physical Health Clinical Improvement Group (CIG) (*n* = 17), including nursing staff (*n* = 6), consultant forensic psychiatrists (*n* = 1), general practitioners (*n* = 2), clinical and health psychologists (*n* = 1), occupational therapists (*n* = 1), healthy lifestyle service lead (*n* = 1), dietitians (*n* = 2), physiotherapists (*n* = 1), exercise practitioners (*n* = 1) and administration staff (*n* = 1).

Additional contributors were engaged through Nursing Leadership Forums (*n* = 29) and the Research forum, representing a broader range of nursing staff in clinical and managerial roles. These figures reflect participation across multiple meetings and may include repeat attendance.

#### 3.2.2 Service Users

Service users participated through established forums, the Service User Research Involvement Groups and the Physical Health Clinical Improvement Group (CIG).

Across the study, six types of pre‐existing forums were used to facilitate PPI engagement, including Healthy Weight CQUIN meetings, Nursing Leadership Forums, Physical Health Clinical Improvement Group (CIG) meetings and Trust Research Forums. These forums were embedded within routine clinical governance structures, and the researcher attended them to present study aims, facilitate structured discussions, and obtain iterative feedback.

Attendance and professional composition across these forums are summarised in Table 1. Attendance figures are based on meeting records and should be interpreted as approximate due to variation across sessions and potential repeat attendance. Variation in attendance reflects the dynamic nature of secure inpatient settings, including shift patterns, clinical priorities, and service user availability. The use of existing consultation structures has been recommended to facilitate meaningful engagement in secure mental health settings (Aboaja et al. 2021). This approach enabled iterative stakeholder involvement while minimising additional burden on staff and service users within resource‐constrained inpatient environments.

**TABLE 1:** Summary of the attendance and professional composition across these forums.

Forum category	Specific forum/Involvement group	Meeting date(s)	Contributors (*n*)	Participant breakdown
Professional Forums	Nursing Leadership Forum	13 Oct 2021; 9 July 2025	29 25	Nursing staff (clinical and managerial)
	Physical Health Clinical Improvement Group (CIG)	13 Oct 2021; 10 May 2023; 16 June 2023	17 18 16	Multi‐disciplinary: Nursing (6), General Practitioners (2), Dietitians (2), Occupational Therapist (1), Physio (1), Psychologist (1), Exercise Team (1), Consultant Psychiatrist (1), Healthy Lifestyle Lead (1), Admin (1)
	Healthy Weight CQUIN Meeting and Follow‐up	14 Sept 2021; 9 Nov 2021	19 13	Multi‐disciplinary team
	Research Forum	11 May 2022; 7 July 2023; 4 Apr 2025	27 14 22	Multi‐disciplinary team and research stakeholders
Service User Forums	Low‐secure Male Service User Research Involvement Group	15 Dec 2023; 26 Sept 2025	10 6	Male service users in low‐secure care
	Medium‐secure Male Service User Research Involvement Group	15 Dec 2023; 18 July 2025	16 14	Male service users in medium‐secure care
	Female Service User Research Involvement Group (Low and Medium)	10 Dec 2021; 12 Jan 2024; 8 Aug 2025	5 8 6	Service users in female secure services

### 3.3 Data Collection and Analysis

Each PPI round used tailored engagement methods. Structured group discussions were conducted across multiple PPI meetings, which were facilitated using topic guides and visual prompts to support inclusive participation and manage power dynamics. The Nominal Group Technique is a structured consensus method designed to elicit and prioritise ideas while minimising power imbalances (Delbecq et al. 1975). It was used in selected rounds to generate and prioritise ideas through independent idea generation, round‐robin sharing, group clarification and ranking to achieve consensus. Think‐aloud usability testing was conducted in paired or small‐group formats, with participants verbalising their thoughts as they reviewed tool drafts, enabling the identification of comprehension, emotional responses and practical barriers. Sessions were facilitated by the researcher with attention to psychological safety, and adaptations (e.g., shorter sessions and informal settings) were made to accommodate the constraints of secure wards.

The collaborative engagement sessions and usability tests were implemented as Patient and Public Involvement (PPI) and co‐design activities rather than a formal, ethically approved research study. Consequently, in line with national health research governance standards, these activities did not yield formal research ‘data’, and verbatim audio transcriptions were not produced. Instead, qualitative feedback was documented across two distinct streams: process‐level insights regarding study design and research procedures, alongside intervention‐specific feedback from both staff and service users to refine the tool, and recorded through detailed research field notes, meeting minutes and thematic logs.

Process‐level PPI insights informed the study's focus and design (e.g., research questions, recruitment and ethical procedures). Intervention‐specific feedback directly informed the content and design of the Nurse‐Led Weight Talk Tool. This feedback was analysed iteratively using the Table of Changes (TOC) method (Yardley et al. 2015) to document modifications across design iterations systematically. Suggested changes were prioritised using the MoSCoW framework (‘Must do, Should do, Could do, Would like to do’). Acceptability and usability were further examined using the Theoretical Framework of Acceptability (TFA) (Sekhon et al. 2017) and the Cognitive Model of Question Response (Tourangeau 1984). Table 2 summarises each PPI round chronologically, mapping the respective contributors, specific engagement techniques, key feedback themes and resulting project decisions. Participant numbers reported across PPI rounds reflect engagement across multiple meetings and may include repeat contributors; therefore, totals should not be interpreted as unique individuals.

**TABLE 2:** Summary of PPI rounds and key outcomes.

PPI round/INVOLVE stage	Timeline	Contributors	PPI activities	Engagement techniques used	Summary of feedback received	Key outcomes
Round 1: Defining and prioritising research questions (Stage 1)	Sept 2021–Mar 2022	Multi‐disciplinary team (*n* = 46)	• Attended nursing leadership forum and physical health CIG. • Service user forum discussions.	Nominal Group Technique for priority setting; structured group discussion; consensus voting.	• Need for consistent, structured intervention to support staff confidence in weight management. • Service users highlighted barriers such as limited autonomy, restricted food choices, and medication effects.	• Refined project direction and prioritised co‐design of a nurse‐led tool to support healthy weight management in secure settings.
Round 2: Refining research questions (Stage 2)	Apr 2022–Sept 2022	Multi‐disciplinary team (*n* = 27)	• Follow‐up PPI meetings, including Healthy Weight CQUIN and Women's Service User Forum.	Guided discussion using person‐centred prompts; iterative feedback cycles.	• Emphasised the need for person‐centred, theory‐informed intervention. • Confirmed value of aligning with Person‐Based Approach (PBA).	• Research aims aligned with the PBA framework. • Refined focus on barriers/enablers of nurse‐led weight management.
Round 3: Developing research proposals (Stage 3)	Oct 2022–Dec 2022	Service users (*n* = 20); nursing staff (*n* = 10)	• Steering group reviewed ethics and participant materials. • PPI contributors advised on interview topic guides and recruitment approach.	Document review; consensus feedback; guided discussion.	• Importance of trust, continuity, and non‐judgmental communication • Concerns about staff confidence and time pressures.	• Participant materials refined • Interview guides finalised • Contextual priorities identified to inform later tool development.
Round 4: Co‐design and intervention development (Stage 4)	Jan 2023–Jan 2024	Service users (*n* = 30); Physical Health CIG members (*n* = 34 across two co‐design sessions)	• Conducted Service User Involvement Group meetings (by gender/security). • Held two CIG sessions to co‐design an intervention.	Facilitated co‐design workshops; visual prompts; structured feedback discussions.	• Service users requested the removal of scales and judgmental language. • Suggested new icons and clearer visual layout. • Recommended inclusion of ‘feeling judged’ statement.	• Tool design modified to improve inclusivity and readability. • Introduced warm‐up/debrief processes for ethical engagement.
Round 5: Analysing Data and Refining the Tool (Stage 5)	Jan 2024–Jun 2024	Nurse–service user dyads (*n* = 19)	• Paired think‐aloud sessions to evaluate usability and acceptability. • Applied TOC and MoSCoW prioritisation methods.	Think‐aloud methodology; warm‐up tasks; structured debriefs; TOC and MoSCoW prioritisation.	• Staff highlighted usability and practicality; requested flexibility in use during sessions. • Service users recommended colour contrast improvement and plain language.	• Refined layout, colour contrast, and phrasing for readability. • Demonstrated high acceptability (coherence, affective attitude, perceived effectiveness).
Round 6: Disseminating findings (Stage 6)	Mar 2025–Sept 2025	Service users (*n* = 26); nursing leadership (*n* = 25)	• Three service user research groups and nursing leadership meeting.	Interactive presentations; structured feedback discussions.	• Staff found the tool helpful in structuring sensitive discussions; requested training video and integration into e‐records. • Service users advised improved colour palette, text contrast, and visibility across wards.	• Positive staff and service user endorsement. • Proposed implementation strategies (community meetings, ward displays, MDT collaboration). • Reinforced the feasibility and sustainability of tool integration.

*Note:* Engagement techniques are briefly described in Section 3 to support transparency and replicability.

## 4 Results

**FIGURE 1:**
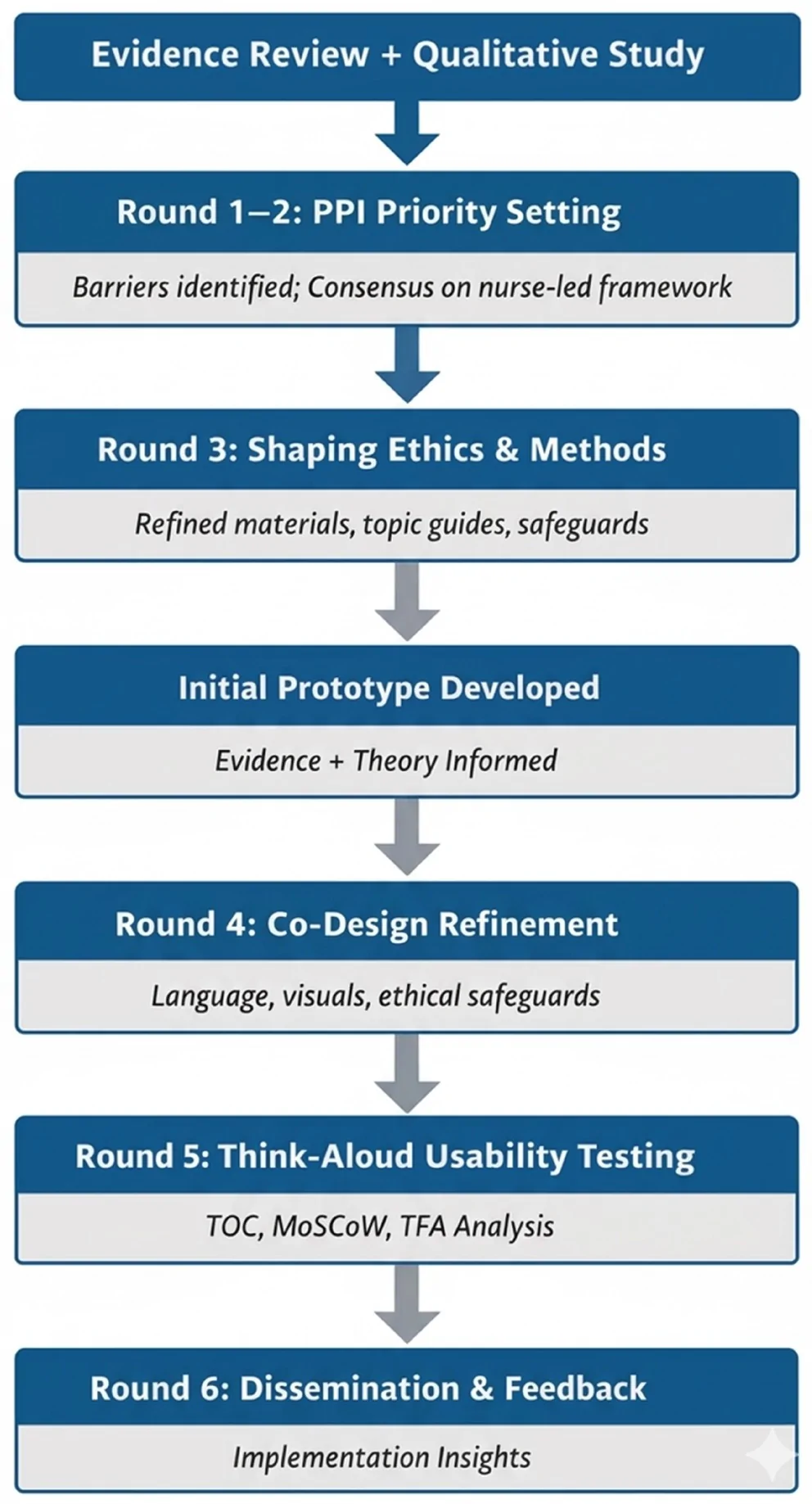
Overview of co‐design and PPI process across study stages.

The following sections summarise key insights from each co‐design and PPI round, illustrating how iterative stakeholder engagement informed the development, refinement and acceptability of the intervention. To support clarity, Figure 1 provides an overview of the co‐design process and its relationship to the study stages, while Table 2 details the specific activities, engagement techniques and outcomes associated with each round.

### 4.1 Round 1–2: Defining and Refining Research Questions

The initial stages of the Public and Patient Involvement (PPI) process focused on identifying priorities for improving weight‐management support in secure settings. Participants, including the multi‐disciplinary team described above, and service users, discussed both structural and motivational barriers to healthy weight management. Key challenges discussed included environmental restrictions, limited food choices, medication‐related weight gain and variability in staff engagement across wards. Using the Nominal Group Technique (James Lind Alliance 2024), participants reached consensus on the need for a structured, nurse‐led framework to support consistent, person‐centred conversations about weight. Follow‐up meetings refined the research focus, aligning it with the Person‐Based Approach (PBA) to ensure that subsequent intervention development would be grounded in lived experience, contextual understanding and theoretical rigour.

### 4.2 Round 3–4: Developing Proposals and Undertaking Research

As outlined in Round 3 of the PPI process (Table 2), a multi‐disciplinary steering group, including service users, reviewed draft ethics documentation, participant information sheets, consent processes, and interview topic guides. This PPI activity ensured that study materials were accessible, transparent, and sensitive to the emotional and contextual challenges of discussing weight in secure settings. Steering group feedback helped refine the framing and sequencing of interview questions, supported appropriate language use, and highlighted areas requiring particular care, such as medication effects and perceived power imbalances in nurse–service user interactions.

Although the subsequent interviews with service users and nursing staff constituted formal research data collection, PPI input shaped how these data were generated and interpreted, including the study's focus and the ethical conduct of data collection. Initial concepts for the Nurse‐Led Weight Talk Tool were developed by the researcher based on existing evidence and theory, with PPI contributing to refining the tool's purpose, scope and acceptability. Findings from the qualitative phase are reported in a separate manuscript currently under review.

**FIGURE 2:**
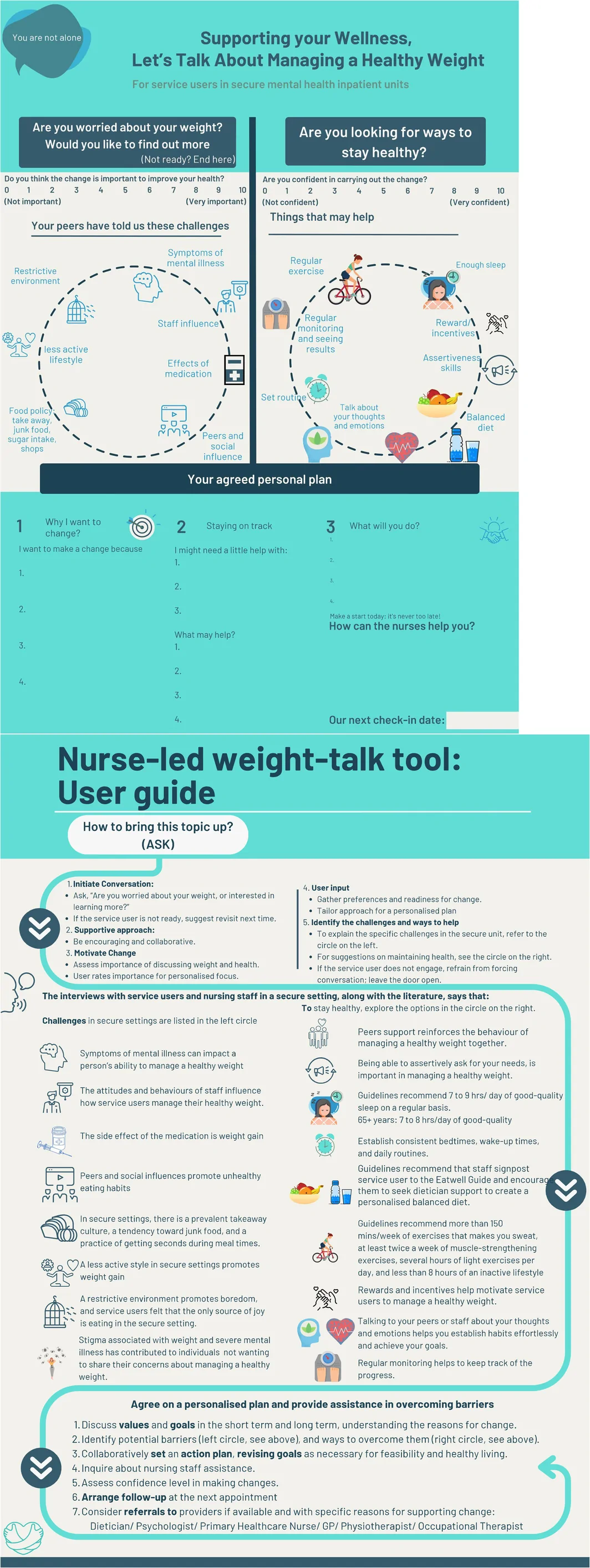
Nurse‐Led Weight Talk Tool (version 1).

Following completion of the evidence review and qualitative study, the research team developed an initial prototype of the Nurse‐Led Weight Talk Tool (Figure 2). The tool was informed by the Person‐Based Approach (Yardley et al. 2021) and relevant behavioural‐change frameworks (Atkins et al. 2017; Michie et al. 2011, 2014). It comprised a brief, structured conversation guide designed to support nurses in initiating and sustaining weight‐related discussions. Specifically, the visual architecture of the tool and its accompanying User Guide were systematically mapped to the COM‐B (Capability, Opportunity and Motivation) domains of the Behavioural Change Wheel (BCW) to address known implementation bottlenecks in inpatient settings. The User Guide targets nurses' Psychological Capability by offering structured, step‐by‐step guidance on how to initiate sensitive conversations (‘ASK’), thereby reducing the communicative dread and stigma often associated with weight‐related discussions. To support service users, the ‘Things that may help’ visual circle provides evidence‐based instructions on lifestyle modification, targeting Physical Capability through explicit instruction on how to perform the behaviour. The left‐hand circle directly operationalises Physical and Social Opportunity by explicitly acknowledging systemic ward‐based barriers (e.g., ward food policy and medication side effects), using them as a collaborative starting point to normalise challenges. Finally, the inclusion of visual 0–10 importance and confidence scales directly targets Reflective Motivation, allowing clinicians to gauge readiness‐to‐change and facilitate self‐determined goal setting without applying clinical pressure. This prototype provided a structured starting point for subsequent PPI‐led refinement.

**FIGURE 3:**
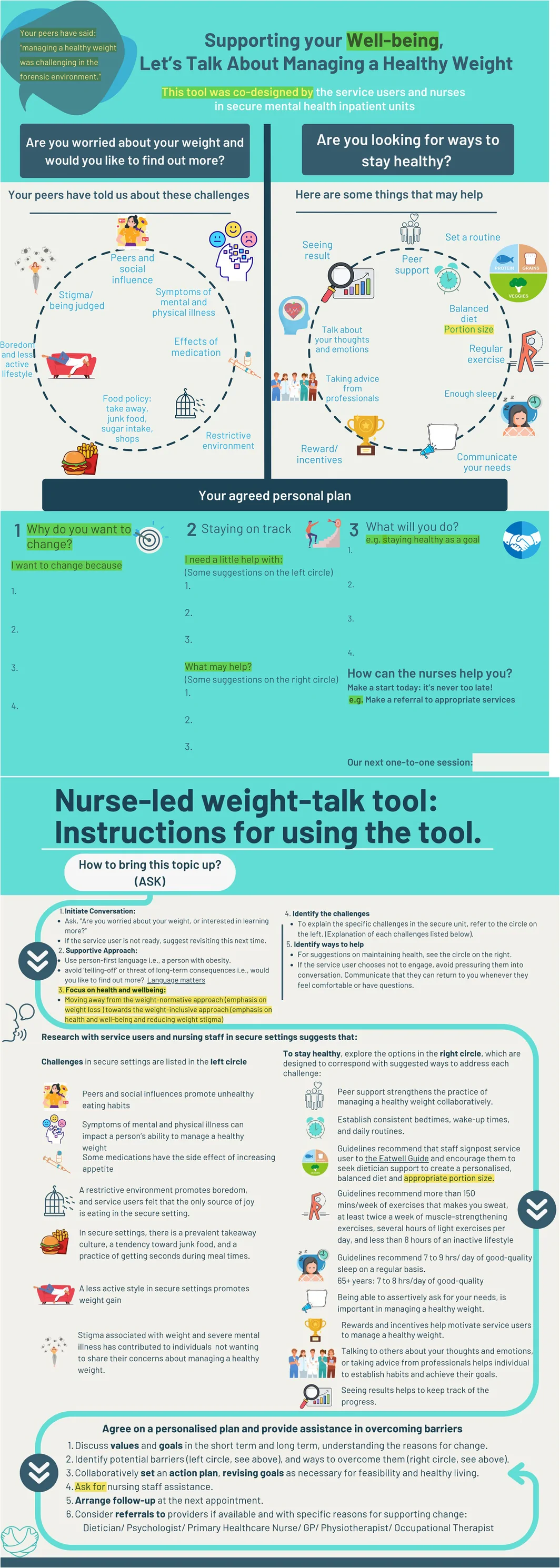
Revised and final version of the Nurse‐Led Weight Talk Tool (version 3). Changes are highlighted in yellow.

Iterative co‐design sessions then focused on refining the tool's structure, language and visual design. The initial prototype (Figure 2) was progressively adapted in response to service user and staff feedback, resulting in a revised version of the Nurse‐Led Weight Talk Tool (version 3; Figure 3). Key changes included the removal of visual readiness scales to reduce clinical pressure and potential stigma. Crucially, the theoretical framing was updated to explicitly pivot from a weight‐normative model (focusing on weight loss) to a weight‐inclusive approach focusing on holistic health, well‐being and stigma reduction. To improve usability and reduce cognitive load during sessions, the personal planning section was restructured to include explicit cross‐referencing prompts, guiding users to draw ‘challenges’ directly from the left visual circle and ‘solutions’ from the right visual circle. Furthermore, terminology was refined to better align with the secure psychiatric environment. This included changing the title focus to ‘Well‐being,’ replacing ‘check‐in’ with ‘one‐to‐one session,’ adding a dedicated speech bubble with forensic‐specific peer quotes to normalise systemic difficulties, and introducing concrete, practical examples (such as targeting ‘staying healthy as a goal’ and prompting for practical nursing referrals). Modifications to the final version are highlighted in Figure 3 to illustrate how PPI directly informed these collaborative, relational and structural design decisions.

Feedback also informed the introduction of ethical safeguards within the co‐design processes, including structured debriefs and flexible scheduling, to accommodate the emotional sensitivity of weight‐related discussions and the operational constraints of secure settings. Collectively, these iterative refinements demonstrate how co‐design enabled the transformation of an initial prototype into a more acceptable, user‐informed intervention, reflecting both clinical and lived experience.

### 4.3 Round 5: Analysing Data and Refining the Tool

Feedback from the co‐design and usability testing phases was analysed using the Table of Changes (TOC) and MoSCoW prioritisation frameworks. Nineteen nurse–service user dyads participated in think‐aloud testing, which provided detailed insights into the tool's usability, clarity and emotional resonance (summarised in Table 2). Guided by the Theoretical Framework of Acceptability (TFA) (Sekhon et al. 2017) and the Cognitive Model of Question Response (Tourangeau 1984), analysis indicated high levels of coherence, perceived effectiveness, and emotional support. When interpreted through the lens of the COM‐B model (Michie et al. 2011), these findings demonstrate that the tool successfully targeted key drivers of professional behaviour change (see Table 2 for a detailed breakdown of resulting design decisions). Nurses reported greater confidence in initiating weight‐related discussions, signalling a shift in their Reflective Motivation as their perceived self‐efficacy and comfort with the topic grew. Furthermore, nurses appreciated the structured format for guiding sensitive conversations, which directly augmented their Psychological Capability by providing clear cognitive protocols and linguistic templates to overcome role ambiguity. Service users described feelings of being ‘listened to’, ‘understood’ and ‘actively involved’ in their care, emphasising that the tool made weight‐related dialogue feel safer and more collaborative.

Crucially, tracking these sessions through the thematic logs and observational field notes highlighted how the tool's structural fidelity could be maintained when navigating highly diverse service user presentations, including varying cognitive capacities, acute symptoms and fluctuating motivational levels. For instance, testing revealed that rigid numeric scales occasionally induced performance anxiety or clinical pressure during acute distress. Consequently, the co‐design team iteratively adapted the tool to feature a weight‐inclusive, ‘opt‐in’ mechanism that prioritised relational safety over metric tracking. To support service users experiencing cognitive slowing or sedation, text‐dense elements were adapted into simplified iconography paired with clear cross‐referencing prompts linking the personal plan back to the challenge and solution circles. These iterative, data‐driven modifications ensured the tool preserved its core behaviour‐change mechanisms while remaining structurally resilient and safe across a broad spectrum of acute clinical needs.

### 4.4 Round 6: Disseminating Findings

Dissemination activities, conducted between March and September 2025, facilitated reciprocal feedback and supported the translation of findings into clinical practice. The Nurse‐Led Weight Talk Tool was presented at Service User Research Groups (18th July 2025, 8th August 2025, 26th September 2025). Attendees (*n* = 26) provided detailed, constructive feedback on the tool's visual presentation, highlighting improvements to colour contrast, iconography, and layout, and proposing practical dissemination strategies, such as displaying posters on ward noticeboards and in gyms, integrating discussions into ward community meetings, and promoting cross‐disciplinary collaboration.

A Nursing Leadership Meeting (*n* = 25) provided senior‐level endorsement of the intervention, with attendees recognising its potential to embed therapeutic non‐judgemental communication into everyday nursing practice. Leaders also recommended developing concise training resources, such as short instructional videos, to support consistent implementation and staff confidence. Collectively, these dissemination activities reinforced the Nurse‐Led Weight Talk Tool's contextual relevance, ownership and sustainability, positioning it for integration into secure inpatient care pathways.

## 5 Discussion

Embedding Public and Patient Involvement (PPI) and co‐design principles throughout the development of the *Nurse‐Led Weight Talk Tool* shaped both the intervention and the research process. The iterative, participatory approach ensured that the final tool was not only theoretically informed but also grounded in the lived experiences of service users and the practical realities of nurses working in secure mental health settings. This section reflects on what co‐design offered, the challenges encountered, and the lessons learned, situating these insights within the broader co‐design literature.

### 5.1 Benefits of co‐Design in Intervention Development in Secure Mental Health Settings

The co‐design approach was instrumental in bridging the gap between evidence and practice and in strengthening the quality of the research process. In contrast to traditional researcher‐led or top‐down approaches (Ramadi et al. 2022), co‐design enabled iterative, contextually responsive development that captured the emotional, relational and operational realities of secure mental health care (Boamah et al. 2025). Crucially, early PPI involvement shaped the design of the data collection methods, supporting the generation of richer, more meaningful qualitative data. Service users advised on the phrasing, sequencing and sensitivity of interview questions, helping to reduce defensiveness and distress around weight‐related discussions, while nursing staff highlighted practical considerations (e.g., timing, setting and workload) that improved feasibility and participant engagement.

As a result, data collection was more attuned to participants' lived experiences and clinical contexts, enhancing rapport, depth of disclosure and relevance of findings. Service users offered critical insight into language, tone and design aesthetics, elements that also informed how questions were framed and explored, while nursing staff ensured that discussions reflected real‐world practice rather than idealised processes. These contributions illustrate a key strength of co‐design: the integration of tacit, experiential knowledge with professional and theoretical expertise to support both high‐quality data generation and intervention development.

Consistent with previous findings (Arumugam et al. 2023; Schouten et al. 2022; Todd et al. 2020), this participatory process enhanced the acceptability, usability and contextual fit of the intervention while promoting trust, collective ownership and sustained engagement—factors that are particularly critical to methodological rigour and implementation success in restrictive clinical settings.

### 5.2 Engagement, Empowerment and Reflective Practice

The Co‐design process fostered genuine collaboration rather than tokenistic consultation. Service users described participation as empowering, offering opportunities to be ‘heard’ and to influence care delivery. This sense of agency aligns with recovery‐oriented principles and is supported by evidence that PPI can enhance empowerment, connection, and well‐being (Jay et al. 2024). Importantly, the co‐design process itself had therapeutic value, providing a structured, respectful space for dialogue about health and self‐image—topics often marginalised in secure settings.

For nursing staff, engagement in co‐design encouraged reflection and critical dialogue around weight stigma, body image, and the nurse's role in promoting physical health within mental health care. These reflective practices mirror findings that co‐design can challenge taken‐for‐granted professional assumptions and support trauma‐informed and recovery‐oriented practice (Lim et al. 2019; Muchamore et al. 2024; Nikopaschos et al. 2023).

In this study, co‐design functioned not only as a developmental approach but also as a cultural intervention by disrupting routine, task‐focused approaches to weight management and legitimising relational, collaborative conversations in a context where autonomy is often constrained (Rogers et al. 2021). Co‐design has been increasingly conceptualised as a mechanism for cultural and normative change in healthcare, enabling shifts in professional identity, decision‐making and power relations rather than simply improving intervention design (Greenhalgh et al. 2016).

This aligns with wider evidence that co‐designed interventions are more likely to reflect cultural, relational and contextual realities of care, rather than focusing narrowly on content or behaviour change alone (Ospina‐Pinillos et al. 2019; Verbiest et al. 2019). As critics note, interventions developed without such participatory engagement risk poor cultural fit and limited real‐world implementation (Simpson and Hoover 2024). By embedding co‐design throughout development, this study produced an intervention that was both culturally sensitive and realistically implementable within secure mental health settings.

### 5.3 Ethical Challenges

Working with service users within secure forensic settings presented distinctive ethical and logistical challenges. Discussions around weight were emotionally charged for service users, intertwined with issues of body image, self‐esteem and trauma (Davies 2021). Institutional restrictions, unpredictable fluctuations in service users' care needs and clinical risk, and staffing constraints limited the flexibility typically afforded in community‐based participatory research (Ige 2022).

Ethical safeguards were therefore embedded throughout the process. Continuous consent, reminders of voluntary participation and structured debrief sessions supported participant wellbeing and mitigated potential distress. These practices reflect the relational ethics framework described by Pidgeon (2019), emphasising ‘5Rs’: respect, reciprocity, responsibility, relevance (in line with the meaning and value of the research context) and reverence (recognising the interrelation with the spiritual realm) in participatory research.

### 5.4 Reflexivity and Researcher Positioning

Ethical reflexivity was central to maintaining rigour and trustworthiness. The researcher engaged in ongoing reflection through field notes and supervision, critically examining how positionality, power dynamics and professional background shaped interactions and interpretations (Moll et al. 2020; Soedirgo and Glas 2020). This reflexive stance fostered sensitivity to institutional hierarchies and participant vulnerability, ensuring that engagement remained authentic and transparent.

### 5.5 Strengths

A key strength of this study was the use of co‐design as a relational methodology rather than solely a technical development strategy. The intervention's most meaningful features emerged through sustained dialogue, negotiation and shared reflection between service users and nursing staff, rather than through methodological innovation alone. This process enabled the integration of experiential knowledge with clinical expertise, producing an intervention grounded in the everyday realities of secure mental health care.

Co‐design functioned as a form of knowledge co‐production, generating insights into how and why weight‐related conversations succeed or falter within restrictive environments, insights unlikely to be captured through researcher‐led methods alone. By attending to relational dynamics, power and communication, the process enhanced contextual fit, acceptability and ethical robustness.

The value of co‐design in mental health research is supported internationally. For example, participatory design in Colombia engaged young people in developing a digital mental health platform, demonstrating that co‐design can produce usable and contextually relevant interventions even outside high‐income settings (Ospina‐Pinillos et al. 2025). Systematic reviews further confirm that co‐design is increasingly adopted globally to enhance engagement, contextual relevance and implementation success across global mental health intervention development, and recognises the importance of involving people with lived experience (Veldmeijer et al. 2023).

Together, these findings reinforce that co‐design is not only a method for improving intervention quality but also a mechanism for advancing person‐centred, inclusive and ethically robust mental health practices across local and international contexts.

### 5.6 Challenges and Lessons for Future Practice

Conducting co‐design and PPI within secure mental health settings revealed a set of interrelated challenges that directly informed key methodological and practice‐based lessons. Central to the process was the need to establish trust in environments where autonomy is constrained and research fatigue can be high. Sustained presence, transparency about decision‐making and clarity regarding how feedback would be used proved essential for fostering meaningful engagement. This reinforces evidence that trust‐building is not a preparatory step but an ongoing requirement for effective co‐design in secure and forensic contexts.

Operational constraints also posed challenges. Staff shortages, variable shift patterns, ward restrictions and competing clinical priorities often limited consistent participation from both nursing staff and service users. In response, the research team adopted flexible and opportunistic strategies, including aligning PPI activities with existing ward community meetings and staff forums, offering multiple engagement points and adapting session timing and format. These adaptations underscore a key lesson for future practice: co‐design in secure settings must remain responsive to fluctuating clinical realities rather than rely on fixed, idealised research schedules. This finding aligns with broader co‐design literature, which emphasises flexibility and responsiveness in complex healthcare systems (McAllister et al. 2021).

Power dynamics between researchers, clinicians and service users required careful and ongoing attention (Kalocsai et al. 2023). The researcher's dual role as academic and facilitator necessitated reflexive awareness of how professional hierarchies and institutional culture might inhibit open dialogue. Strategies such as small group discussions, open‐ended questioning and the use of visual prompts were effective in flattening hierarchies and supporting more equitable participation. These approaches highlighted that psychological safety is a precondition for authentic co‐design, particularly when addressing sensitive topics such as weight, stigma and health behaviours.

Several additional lessons emerged through responding to these challenges. Gender‐specific PPI groups, for example, enabled more open discussions and revealed nuanced differences in experiences of weight stigma and engagement, directly shaping the intervention's tone and framing. Similarly, balancing inclusivity with methodological rigour required transparent decision‐making when feedback diverged or when attendance was inconsistent. The use of structured processes, such as the Table of Changes (TOC) method, supported accountability by ensuring that all feedback was documented, considered and clearly justified.

Overall, these challenges highlight that co‐design in secure mental health care is neither linear nor neutral. While nurse‐service user dyad discussions generated rich, practice‐relevant insights, they also presented limitations. Power imbalances, concerns about disclosure and the presence of a known clinician could constrain service users' willingness to express discomfort or critique aspects of care. Similarly, some nursing staff may have moderated their responses due to professional identity or perceived scrutiny. These dynamics required careful facilitation, clear boundaries around confidentiality and ongoing attention to psychological safety. Co‐design in secure settings, therefore, demands time, institutional support, ethical reflexivity and methodological flexibility. The lessons learned underscore that meaningful PPI is achieved not by avoiding constraints but by actively working with them to transform practical and ethical challenges into opportunities for more inclusive, contextually grounded intervention development.

## 6 Conclusion

This study asked how co‐design and Patient and Public Involvement (PPI) can inform the development of a nurse‐led weight management intervention within secure inpatient mental health settings. Findings demonstrate that embedding co‐design across the research cycle enabled service users and nurses to collaboratively shape priorities, methods and intervention refinement, ensuring relevance to everyday nursing practice and the relational realities of secure care.

Importantly, co‐design functioned not only as a methodological approach but as a nursing practice in itself, supporting dialogue, shared decision‐making and ethical reflexivity when addressing sensitive health topics. The iterative processes described illustrate how co‐design can strengthen acceptability, enhance contextual fit and foster shared ownership in environments characterised by complexity, restriction and workforce pressures.

These insights are highly relevant to international mental health nursing contexts where nurses play a central role in delivering holistic, person‐centred care under similar institutional constraints. Co‐design offers a transferable framework for developing and adapting interventions that are culturally sensitive, ethically robust and sustainable across diverse secure and inpatient settings. Future mental health nursing research should continue to evaluate co‐design not only for its outcomes, but for its capacity to support inclusive, relational and practice‐informed innovation.

### 6.1 Relevance for Clinical Practice

This study positions co‐design as a transferable and practice‐relevant methodology for developing interventions in secure mental health settings internationally. Evidence from Australia, Canada, the United States and the Netherlands demonstrates that participatory approaches enhance the acceptability, cultural relevance and sustainability of mental health interventions across diverse service systems (Bell et al. 2023; Moghimi et al. 2025; Schouten et al. 2022). Consistent with this, engaging service users and nursing staff throughout this study embedded both practical and emotional considerations into intervention development, strengthening contextual fit and implementation readiness (Hawke et al. 2024).

Co‐design also supports reflective nursing practice and enables engagement with sensitive topics, an ongoing challenge across forensic and inpatient mental health services globally (Almomani et al. 2023). International research indicates that a collaborative approach can reduce power imbalances, enhance therapeutic relationships, and improve staff confidence when addressing complex issues such as physical health stigma and autonomy (Arumugam et al. 2023; Todd et al. 2020). In this study, co‐design illuminated nuanced differences in perspective across gender, security level and lived care experience, reinforcing the importance of culturally and contextually responsive nursing practice.

For nurse researchers and clinical leaders worldwide, embedding co‐design within intervention development or adaptation may improve research relevance, strengthen data credibility and increase the likelihood of successful implementation. However, international evidence consistently indicates that meaningful co‐design requires organisational commitment, protected time, leadership endorsement and strategies to safeguard psychological safety (Slattery et al. 2020; Schouten et al. 2022). Structured engagement techniques, iterative feedback cycles, visual prompts and explicit attention to relational power dynamics may facilitate these processes across varied healthcare systems.

When adequately supported, co‐design offers a robust framework for advancing person‐centred, culturally sensitive and ethically grounded mental health nursing practice in secure and restrictive care environments globally.

## Author Contributions

All authors meet the criteria for authorship as defined by the International Committee of Medical Journal Editors. Novia K.N. Mak: conceptualisation, study design, data collection, facilitation of PPI activities, data analysis, interpretation of findings, and manuscript drafting. Bridget Dibb, Nicholas Stokes, Emerita Elizabeth Alexandra Barley and Magdalena Zasada: supervision, methodological guidance, contribution to study design, critical revision of the manuscript for intellectual content. All authors have read and approved the final manuscript and agree to be accountable for all aspects of the work.

## Funding

The authors have nothing to report.

## Disclosure

The authors alone are responsible for the content and writing of this article.

## Conflicts of Interest

The authors declare no conflicts of interest.

## Data Availability

The data that support the findings of this study are available from the corresponding author upon reasonable request.
